# Natural Products Targeting the Mitochondria in Cancers

**DOI:** 10.3390/molecules26010092

**Published:** 2020-12-28

**Authors:** Yue Yang, Ping-Ya He, Yi Zhang, Ning Li

**Affiliations:** Inflammation and Immune Mediated Diseases Laboratory of Anhui Province, School of Pharmacy, Anhui Medical University, Hefei 230032, China; yangyue9288@163.com (Y.Y.); hepingya2013@163.com (P.-Y.H.); zhangyi20201125@163.com (Y.Z.)

**Keywords:** natural products, mitochondria, cancer, cell death

## Abstract

There are abundant sources of anticancer drugs in nature that have a broad prospect in anticancer drug discovery. Natural compounds, with biological activities extracted from plants and marine and microbial metabolites, have significant antitumor effects, but their mechanisms are various. In addition to providing energy to cells, mitochondria are involved in processes, such as cell differentiation, cell signaling, and cell apoptosis, and they have the ability to regulate cell growth and cell cycle. Summing up recent data on how natural products regulate mitochondria is valuable for the development of anticancer drugs. This review focuses on natural products that have shown antitumor effects via regulating mitochondria. The search was done in PubMed, Web of Science, and Google Scholar databases, over a 5-year period, between 2015 and 2020, with a keyword search that focused on natural products, natural compounds, phytomedicine, Chinese medicine, antitumor, and mitochondria. Many natural products have been studied to have antitumor effects on different cells and can be further processed into useful drugs to treat cancer. In the process of searching for valuable new drugs, natural products such as terpenoids, flavonoids, saponins, alkaloids, coumarins, and quinones cover the broad space.

## 1. Introduction

Cancer is a threat to human health and is the leading cause of premature death; thus, it reduces the productivity of a country. Cancer rates are rising, driven by unhealthy lifestyles, business interests, and an aging society. According to the Global Cancer Observatory (GLOBOCAN) 2018 database, compiled by the International Agency for Research on Cancer, 18.1 million people were diagnosed with cancer and 9.6 million died in 2018. The most common types of diagnosed cancers are lung cancer, female breast cancer, prostate cancer, colorectal cancer, stomach cancer, and liver cancer [1,2]. Despite tremendous efforts to implement new cancer chemotherapy methods, cancer remains a major problem worldwide. Therefore, it is necessary to find new therapeutic drugs that have specific effects on various cancer cells.

Natural products are important sources of lead compounds and new drugs, which include the components or metabolites of plants, animals, insects, marine organisms, microorganisms, as well as many endogenous chemical constituents in humans and animals [3]. Natural products also include water or alcohol extracts of plants, animals, and fungi, etc. [4,5]. This article mainly discusses individual compounds. In drug discovery and development, natural products have played an important role, especially for anticancer drugs. A great quantity of anticancer medicines are natural products or derivatives of them [6]. Taxol, isolated from *Taxus baccata*, is the most successful antitumor drug that has been found. It has been widely used in the clinical treatment of breast cancer, ovarian cancer, some head and neck cancers, as well as lung cancer [7,8]. Vincristine is another great anticancer natural product, derived from *Catharanthus roseus,* and often used for the treatment of acute lymphocytic leukemia [9,10]. Natural products, as a source of anticancer drugs, are a vast area worth exploring.

## 2. The Role of Mitochondria in Cancer Cells

**Figure 1:**
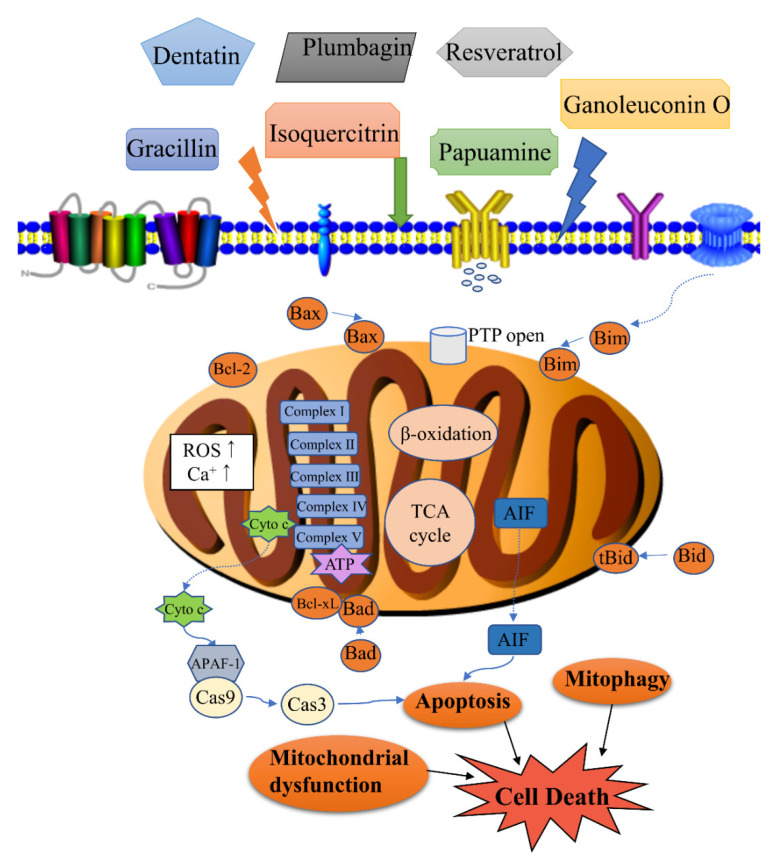
Modulation of mitochondrial-related cell death by natural products. Cell death associated with the activity of natural products includes apoptosis, mitophagy, mitochondrial dysfunction, etc. Apoptosis is regulated by the levels of Bcl-2 (B-cell lymphoma-2) family proteins, release of cytochrome c, and caspase activation. Mitophagy is the targeted phagocytosis and destruction of mitochondria by the autophagy machinery, and it is generally considered as the main mechanism of mitochondrial quality control. A decrease in energy production, an increase of reactive oxygen species (ROS) and permeability transition pore (PTP) opening can lead to mitochondrial dysfunction.

Mitochondria are energy-producing structures and the main site for aerobic respiration in cells, and are therefore called the “powerhouse of the cell” [11,12]. Mitochondria are associated with many diseases, such as Parkinson’s disease [13], diabetic nephropathy [14], acute kidney injury [15], and Down syndrome [16]. Mitochondria also play an important role for cell signaling, apoptosis regulation, and energy metabolism in drug-induced cancer cells death; therefore, they are considered a significant target in cancer chemotherapy [17]. Some scholars have reviewed the mitochondrion as a target of anticancer therapy over the years [18,19,20,21]. Moreover, modulation of mitochondrial-dependent pathways by natural compounds is diverse (Figure 1). However, few researchers have reviewed natural products that regulate mitochondrial pathway in cancers.

## 3. Mitochondrial Control of Apoptosis

Mitochondrial involvement is an important pathway in the process of apoptosis. The Bcl-2 protein family regulates apoptosis by controlling mitochondrial permeability. Anti-apoptotic proteins B-cell lymphoma-2 (Bcl-2) and B-cell lymphoma-extra large (Bcl-xL) reside in the outer membrane of mitochondria and inhibit the release of cytochrome c. Pro-apoptotic proteins Bax, Bad, Bid, and Bim can reside in the cytoplasm, translocating to mitochondria after receiving a death signal, and promote cytochrome c release into the cytoplasm. Released cytochrome c binds to apoptotic protease activating factor-1 (Apaf-1) to form apoptosome, amplifying the apoptotic cascade [22,23,24].

Necrotic stimulation leads to increased mitochondrial Ca^2+^ uptake and ROS production. High levels of Ca^2+^ and ROS induce the opening of the Cyclophilin-D (Cyp-D) sensitive permeability transition pore (PTP), leading to matrix swelling and Ca^2+^ release. Swelling damages the outer membrane and releases Ca^2+^ activating proteases, phosphatases, and nucleases, leading to necrotic degradation [12].

Fission or fusion rates may change under different growth conditions, and result in an increase or decrease in the number of mitochondria. When mitochondria become damaged, their connectivity is reduced, and mitochondria become shorter and rounder. The change from highly branched to fragmented morphologies may be induced by altered fission or fusion rates. At the early stage of apoptosis, the transition from a mitochondrial network to vesicular punctiform mitochondria was detected [25]. Mitochondrial fragmentation occurs in parallel to the formation of apoptotic bodies, increasing the number of the terminal deoxynucleotidyl transferase dUTP nick end labeling (TUNEL) positive nuclei and cleavage of the caspase substrate polymerase (PARP) [26].

## 4. Mitochondrial Control of Energy Metabolism

Mitochondria provide considerable flexibility for the growth and survival of tumor cells, and play a key role in harsh conditions, such as nutrient depletion and hypoxia. The rapid proliferation of cancer cells requires more mitochondria than normal cells. Therefore, the development of chemotherapeutic drugs for mitochondria is a breakthrough in the fight against cancer. Many scholars have clarified that the mechanical drive of mitochondrial respiration involves the tricarboxylic acid (TCA) cycle, and fatty acid β-oxidation enzymes in the mitochondrial matrix that generate electron donors to fuel respiration and electron transport chain (ETC) complexes, and ATP synthase in the inner mitochondrial membrane (IMM) that carry out oxidative phosphorylation [27]. Some natural products inhibit electron transport chain complexes. Four such complexes are NADH-ubiquinone reductase(complex I), succinate-ubiquinone reductase (complex II), ubiquinol-cytochrome c reductase (complex III), and cytochrome c oxidase (complex IV) [28]. Complex V, which is called ATP synthase, together with the above four complexes, completes oxidative phosphorylation to produce ATP. Inhibition of mitochondrial ETC complex activity can lead to significant mitochondrial dysfunction.

Cardiolipin, which consists of two phosphatidyl residues linked by a glycerol bridge, is a unique phospholipid dimer in the inner mitochondrial membrane in all eukaryotes. Cardiolipins play an important role in preserving mitochondrial structure and function. They support membrane dynamics and stabilize the lateral organization of protein-rich membranes in mitochondria [29]. Cardiolipins are involved in mitochondrial cristae morphology and stability [30], mitochondrial quality control, and dynamics by fission and fusion [31,32] and mitophagy [33]. They can also serve as a binding platform to recruit apoptotic factors in the apoptotic process [34,35]. However, it is still not clear how these events are interconnected and cooperate. In addition, cardiolipins are very susceptible to damage from ROS because of their high content of unsaturated acyl chains. Thus, the stability and function of mitochondria can be impaired by the biophysical properties of the membranes that are altered [36].

In this paper, we attempt to summarize the mechanisms through which natural products exert anticancer effects, as published in the past five years, by using a structural classification, with emphasis on the molecular mechanisms of mitochondrial involvement. Through all the reports, we found that most natural products regulate a series of proteins, such as Bax, Bcl-2, and caspases-3 and -9. Moreover, inhibitors of electron transport chain complexes can also exert anticancer activity. Details can be found in Table 1.

## 5. Natural Products Induce Cancer Cell Death through a Mitochondrial Pathway

### 5.1. Terpenoids

**Figure 2:**
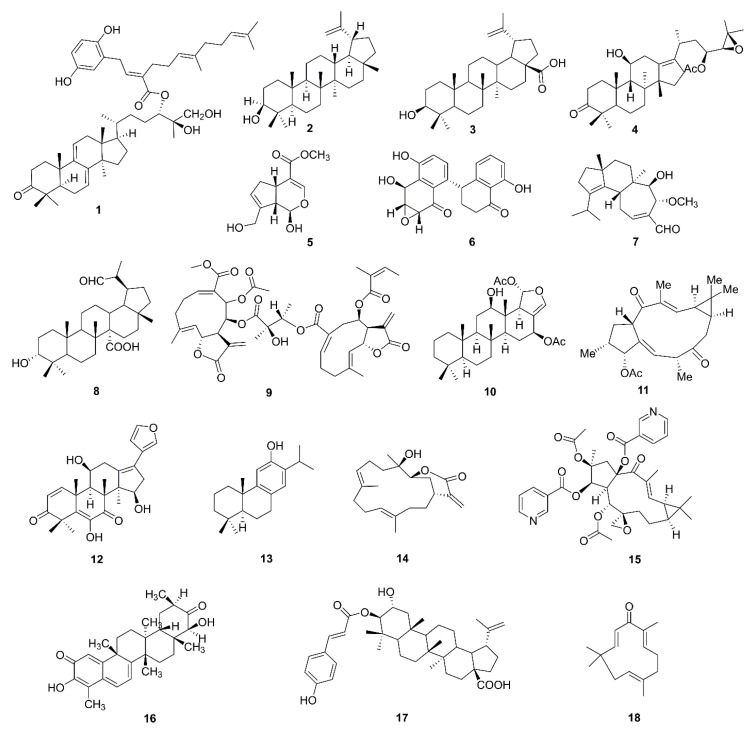
Chemical structures of terpenoids (**1**–**18**).

Terpenoids represent a diverse group of compounds consisting of isoprene or isopentane units linked by various connections. Terpenoids have a wide variety and complex structure throughout the plant kingdom, including monoterpenes (myrcene), sesquiterpenes (artemisinin, gossypol), diterpenes (triptolide), triterpenes (oleanolic acid), tetraterpenes (β-carotene), and polyterpenoids (gutta-percha). Due to their different properties, physiological activities are varied, such as anti-malarial [37], anti-fertility [38], insecticidal activities [39], etc. Here, we will summarize the mechanisms through which terpenoids regulate mitochondrial function to stimulate anticancer effects that have been discovered in recent years (Figure 2).

Ganoleuconin O (GL22) (**1**), a triterpenoid, is obtained from *Ganoderma leucocontextum*. After liver cancer cell line Huh7.5 was treated with GL22, it was observed by transmission electron microscopy that the shape and size of mitochondria were changed, and mitochondrial cristae were fragmented. ATP production of Huh7.5 cells with GL22 treatment was decreased in a dose- and time-dependent manner. The amount of cardiolipin, which has vital structural and metabolic functions in mitochondria, was also decreased. The levels of P53 and Bax were upregulated, while Bcl-2 was downregulated. The dissipation of mitochondrial membrane potential (MMP) resulted in release of cytochrome c from mitochondria to the cytosol and caspase-9 activation, eventually triggering apoptosis [40].

Lupeol (lup-20(29)-en-3β-ol) (**2**), a pentacyclic triterpenoid, is found in fruits, such as strawberries and grapes, and medicinal plants, such as *Bombax ceiba*. It could affect viability of renal cell carcinoma SK-RC-45 cells by altering mitochondrial dynamics. The study showed the lupeol tilted mitochondrial dynamics towards fission in a dynamic balance between fusion and fission, which ultimately led to apoptosis. Mitochondrial morphometric parameters were evaluated by Fiji (ImageJ v1.52e) using the MiNA macro. It was observed that the defined morphological properties networks, mean length, mean network size, and mitochondrial footprint, were decreased in lupeol treated cells compared to control. In addition, anti-apoptotic protein Bcl-2 knockout enhanced the effect of Lupeol, causing mitochondrial fission and cell death [41].

Betulinic acid (BetA) (**3**), a lupane-type triterpenoid, derived from *Betula alba* and other plants has been described to kills tumor cells depending on mitochondrial permeability transition-pore opening. A study found that BetA induced changes of mitochondrial morphology in HeLa cells. The saturation level of cardiolipin can be affected rapidly and directly by BetA. Cardiolipin can regulate mitochondria-dependent cell death with important structural and metabolic functions. Because cardiolipin saturation in mitochondria was enhanced, the mitochondria underwent ultrastructural changes, and then cytochrome c was released inducing cell death [42]. Another study reported that BetA induced production of ROS and decline of MMP in HeLa cells. The protein expression of Bax and caspase 9 was increased. The results indicated ROS was the key factor for regulating the mitochondrial pathway of apoptosis [43]. Wang et al. reported that ROS was increased, MMP was lost, cytochrome c was released, and caspase-3 was activated after treatment with betulinic acid in PC12 cells, while the apoptosis could be reduced significantly by treating with antioxidants [44]. Similarly, Yang et al. reported that betulinic acid induced mitochondria-mediated apoptosis with downregulation of Bcl-2, ROS production and MMP loss in 786-*O* and ACHN renal cancer cells [45].

Alisol B-23-acetate (**4**), a tetracyclic triterpenoid, is a compound from *Alisma orientale*. In human lung cancer NCI-H292 and A549 cells, it reduced MMP, increased ROS level, and the Bax/Bcl-2 ratio. Caspase-3, caspase-9 and PARP were cleaved. Furthermore, cytochrome c was released into the cytoplasm and apoptotic inducing factor was translocated into nuclei [46].

Genipin (**5**) from *Gardenia jasminoides* was applied as an inhibitor of proton transport mediated by mitochondrial uncoupling protein 2 (UCP2). The scholars indicated that after treatment with genipin, dicarboxylate carrier was activated and activity of UCP1, UCP3, and complex III were decreased. UCP2 was inhibited in planar lipid bilayer membranes reconstituted with recombinant UCP2 or isolated mitochondrial proteins from N18TG2 cells [47].

Alternol (**6**), a fermentation product of a microorganism found in the bark of the yew tree [48], reduced the levels of mitochondrial respiration, isocitric acid, fumaric acid, and malic acid. Alternol also remarkably decreased ATP production in PC-3 prostate cancer cells in vitro and in xenograft tissues [49].

Cyathin Q (**7**), derived from *Cyathus africanus*, regulated proteins of the Bcl-2 family, increased ROS generation, and released cytochrome c in HCT116 cells [50]. These anticancer mechanisms may also be used by the compounds 3α-hydroxy-19α-hydrogen-29-aldehyde-27-lupanoic acid (**8**) [51] and uvedafolin (**9**) [133]. Heteronemin (**10**), a secondary metabolite in the sponge *Hippospongia* sp. could induce ROS production in Molt4 cells [134].

Jatrogossone A (**11**), found in *Jatropha gossypiifolia*, is a special class of macrocyclic compound featuring a *trans*-bicyclo [10.3.0] pentadecane framework. It was reported that it affected MMP and induced ROS generation in KONP-8 human leukemic cells, while ROS generation was minimal in non-cancer cells [135]. A limonoid small molecule Walsuronoid B (**12**), isolated from *Walsurarobusta* increased the level of ROS generation and induced mitochondrial and lysosomal dysfunction in Bel-7402 and HepG2 liver cancer cells [136].

There are many other compounds isolated from natural products that exert anticancer effects through regulating mitochondria. They induce production of ROS and reduce the expression of Bcl-2 in various tumor cells. These compounds include ferruginol (**13**) [52,53,137], lobocrassin B (**14**) [53], aellinane (**15**) [54], tingenin B (**16**) [55], 3-*O*-*trans*-p-Coumaroyl alphitolic acid (**17**) [56], and zerumbone (18) [57,58].

### 5.2. Flavonoids

**Figure 3:**
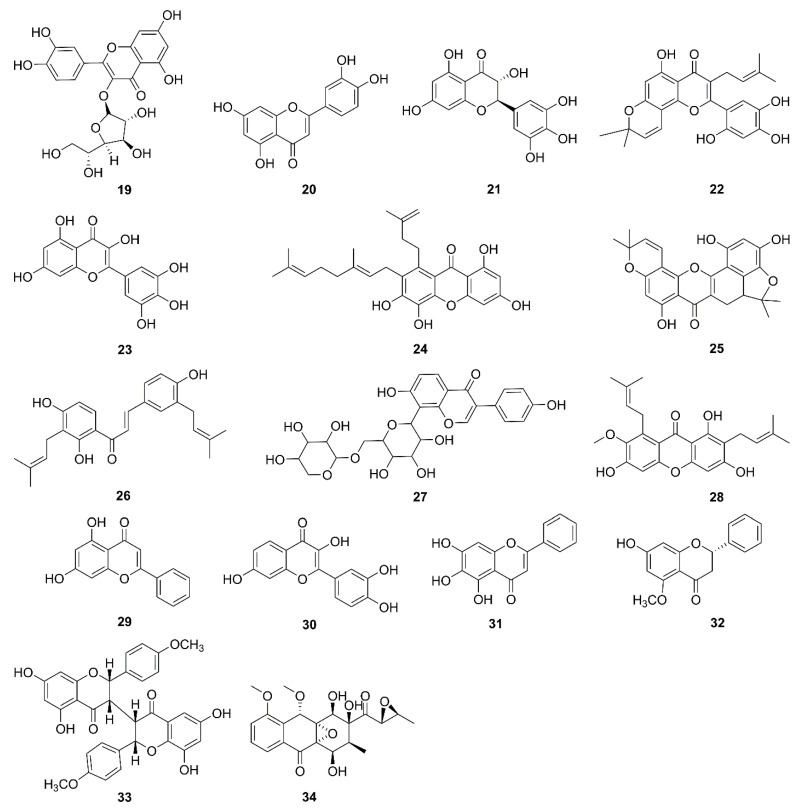
Chemical structures of flavonoids (**19**–**34**).

Flavonoids are compounds that exist widely in nature—in vegetables, fruits [59], and Chinese medicine. The basic backbone of flavonoids is two benzene rings connected by three carbon atoms. Flavonoids are divided into flavones, flavonols, isoflavones, flavanols, anthocyanidins, and isoflavones [60]. Flavonoids have a wide range of health benefits, such as gut health [61], and antioxidant, anti-neuroinflammatory [62], and anticancer [63] properties. They have been developed for use in nutraceuticals, cosmetics, and medical drugs. Chemical structures of flavonoids in recent research are displayed in Figure 3.

Isoquercitrin (**19**), a flavone-based natural product, induced the expression of key proteins in the mitochondrial-mediated apoptosis pathway, and it also caused apoptosis in the breast cancer cell line MDA-MB-231 by inhibition of lysine-specific demethylase 1 (LSD1), which can regulate mitochondrial functions [64,65,66], and has recently become a therapeutic target for cancer. The study found the mitochondrial transmembrane potential and ratio of Bcl-2/Bax was lower in the isoquercitrin + LSD1 siRNA-treated group than in the control group and the LSD1 siRNA-treated, isoquercitrin groups [67,68].

Luteolin (**20**), a dietary compound, can be found in fruits and vegetables including cauliflower, peanuts, and carrots. A study reported it was an inhibitor of Bcl-2 by using structure-based virtual ligand screening. The result of a three-dimensional (3D) molecular docking model showed it has a significant ability to interact with the key residues in the hydrophobic pocket of the Bcl-2 protein. Through the microscale thermophoresis (MST) experiment in SW1990 cancer cells, the K_d_ value of BH3 peptide bound to Bcl-2 was lower than that of luteolin bound to Bcl-2. Besides, luteolin did not bind to the BH3 domain of Bax [69].

Dihydromyricetin (**21**), a plant flavonol, isolated from *Ampelopsis grossedentata*, induced apoptosis in HepG2 cells through a mitochondrial pathway in a recent report. Expression of proteins Bax and Bad was upregulated. The phosphorylation of Bad at Ser112 and Ser136 was inhibited. Expression of Akt and its phosphorylation at Ser473 were reduced. The authors concluded that HepG2 apoptosis might be induced by dihydromyricetin by inhibiting the Akt/Bad signaling pathway and stimulating mitochondrial apoptotic pathways [70,71].

There are some other compounds that induce cancer cell death or an anti-proliferative effect, such as artonin E (**22**) [72], myricetin (***23***) [73], xanthones (**24**) [138], cycloartobiloxanthone (**25**) [139], paratocarpin E (**26**) [74], and puerarin 6′’-*O*-xyloside (**27**) by regulating the Bcl-2 family proteins [75]. Moreover, others induced the overproduction of ROS, such as α-mangostin (28) [76], chrysin (29) [77,78], and fisetin (**30**) [79,80].

A strong antitumor ability was suggested by the release of cytochrome c into the cytoplasm in the combination treatment with baicalein (**31**) and taxol in A2780 cells [81,82]. Alpinetin (**32**), mainly from zingiberaceous plants, increased the resistance of A549 lung cancer cells to *cis*-diammineddichloridoplatium. It regulated the expression of Bcl-2 family proteins, XIAP and cytochrome c [83,84]. Chamaejasmin B (**33**), from *Stellera chamaejasme* exerted an anti-multidrug resistance effect by regulating Bcl-2/Bax ratio, MMP loss, and release of cytochrome c [86]. Mensacarcin (**34**), extracted from *Streptomyces* bacteria could quickly disturb mitochondrial function and energy production [87].

### 5.3. Saponins

**Figure 4:**
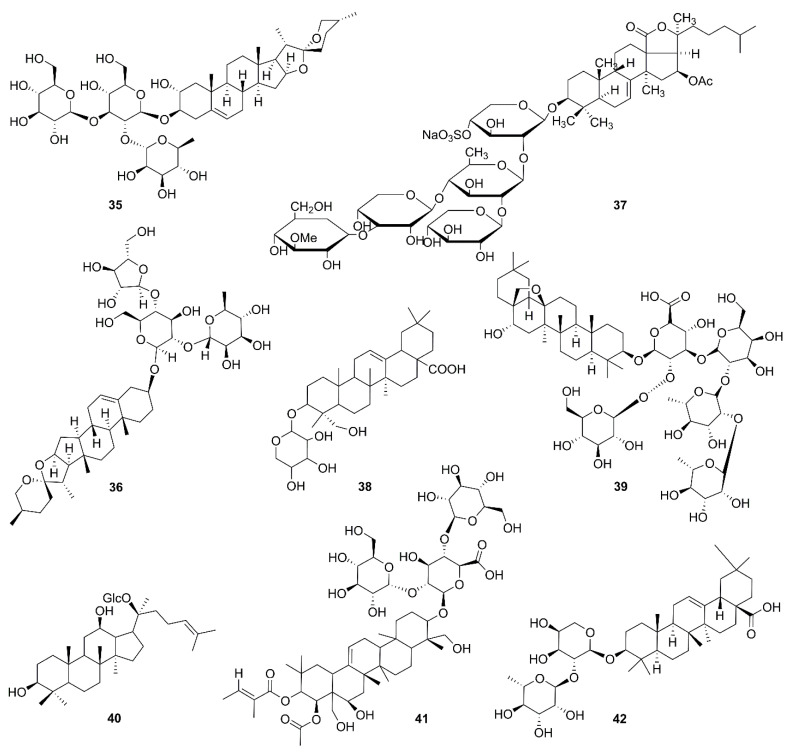
Chemical structures of saponins (**35**–**42**).

Saponins are a kind of complex glycoside synthesized in the plant kingdom, and are composed of sapogenin and sugar chain(s). The saponins can be divided into two groups according to the structure of their sapogenin: triterpenoid or steroidal saponins [88]. Triterpenoid saponins are mainly distributed in Araliaceae, Leguminosae, Campanulaceae, and other plants. Steroidal saponins are commonly reported in Liliaceae, Dioscoreaceae, Amaryllidaceae, etc. [85]. Chemical structures of saponins in recent research are displayed in Figure 4.

Gracillin (**35**), a diosgenin glycoside, is a steroidal saponin. Hye-Young Min et al. reported it exerted anticancer ability by targeting mitochondrial complex II in H226B and H460 cells. Thus, it reduced mitochondria-mediated cellular bioenergetics by inhibiting ATP synthesis and ROS production. It inhibited complex II function by disabling succinic dehydrogenase activity without affecting the succinate: quinone reductase. The cell death induced by gracillin was enhanced by thenoyltrifluoroacetone or 3-nitropropionic acid, which inhibited complex II by binding to the succinate dehydrogenase complex subunit A (SDHA) active site, or the ubiquinone binding site, respectively [89].

Polyphyllin I (**36**), a steroidal saponin, extracted from *Paris polyphylla* rhizomes was reported to induce MDA-MB-231 cells apoptosis through regulating mitochondrial PTEN (Phosphatase and tensin homolog deleted on chromosome ten)-induced kinase 1 (PINK1) levels. PINK1 is localized at the mitochondria as it contains a mitochondrial targeting sequence. Polyphyllin I induced mitochondrial translocation of dynamin-related protein 1 (DRP1) by dephosphorylation of DRP1 at the Ser637 site, resulting in mitochondrial fission, release of cytochrome c, and finally cell apoptosis. It also enhanced stability of the full-length PINK1 on the mitochondrial surface, resulting in the recruitment of microtubule-associated protein light chain 3 beta (LC3B-II), ubiquitin, P62, and PARK2 (a RING domain-containing E3 ubiquitin ligase that can be activated through autoubiquitination) to mitochondria for mitophagy. The knockdown of PINK1 significantly inhibited the mitophagy induced by polyphyllin I and enhanced mitochondrial fission and apoptosis [90,91].

Frondoside A (**37**), a triterpene glycoside, is a marine product first exacted from *Cucumaria frondosa*. After treatment of frondoside A in multiresistant CA46 cells, levels of antiapoptotic Bcl-2 and survivin were decreased. Apoptosis-inducing factor, HtrA2/Omi and cytochrome c were released. It induced production of ROS. Frondoside A targeted mitochondria, which was not dependent on p53 and caspases [92].

*Clematis* hederagenin saponin (hederagenin 3β-*O*-α-l-arabinopyranoside, (**38**) is a triterpenoid saponin of *Clematis ganpiniana*, which was reported to induce apoptosis through the mitochondrial pathway with release of cytochrome c and Apaf-1 and activation of caspase-9 and caspase-3 [93]. In addition, sakuraso-saponin (**39**) from *Aegiceras corniculatum* could regulate expression of Bcl-xL [94,95]. Ginsenoside compound K (**40**) [96] and escin (**41**) [140] induced ROS-mediated apoptosis, and α-Hederin (**42**) from *Hedera helix* induced mitochondrial apoptosis through blocking the NF-κB signaling pathway through the regulation of the levels of Bcl-2, Bax, and cytochrome c [141,142].

### 5.4. Alkaloids

**Figure 5:**
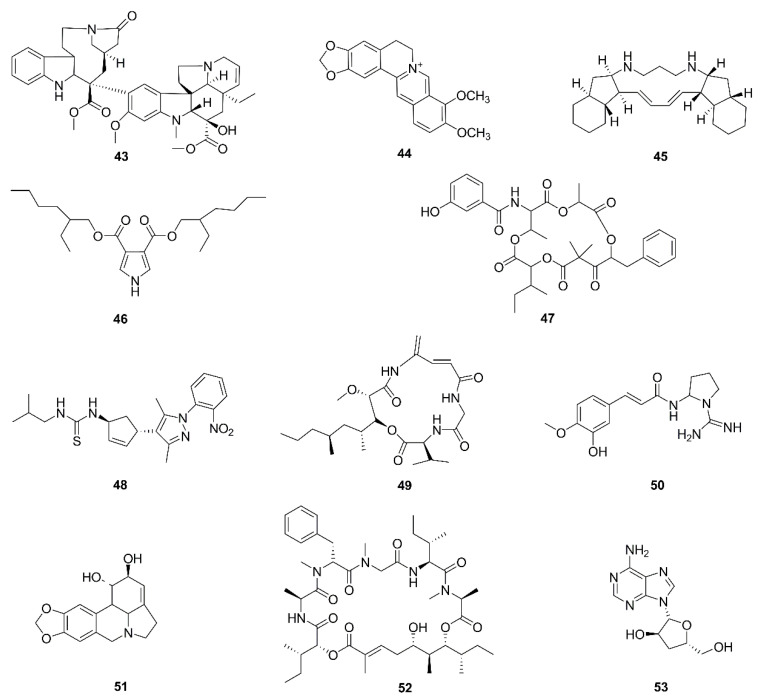
Chemical structures of alkaloids (**43**–**53**).

Alkaloids generally refer to a class of nitrogen-containing natural products, most of which have complex heterocyclic structure, physiological activity, and alkalinity. Morphine isolated from opium has an analgesic effect. Codeine has antitussive effects. Ephedrine has an antiasthmatic effect. Berberine has antibacterial and anti-inflammatory effects. Chemical structures of alkaloids in recent studies are displayed in Figure 5.

Berberine (**44**), extracted from *Rhizomacoptidis*, has long been used as an antimicrobial agent with antitumor abilities in China [97]. In glioma cells, berberine could inhibit the aerobic oxidation and reduce the energy production efficiency of mitochondria, and reduce the metabolic activity by decreasing the activity of extracellular signal-regulated kinase 1/2 (ERK1/2). After treatment with berberine, the ridges and membrane of mitochondria were damaged, the level of ATP dropped rapidly, the ROS scavenger l-Glutathione (GSH) decreased, and NADPH decreased. The authors indicated that inhibition of ERK1/2 activity induced mitochondrial dysregulation by the reduced abundance of p-ERK after treatment of T98G cells [98].

Papuamine (**45**) is a pentacyclic alkaloid extracted from marine natural products including *Haliclona sp*. Intracellular ATP was depleted by papuamine through causing dysfunction of mitochondria in H1299 lung cancer cells as MMP was lost and production of mitochondrial superoxide was increased. The study suggested papuamine, by causing mitochondrial dysfunction, thus reducing the generation of cellular energy, induced cell apoptosis, so as to exert its anticancer effect [99].

Cathachunine (**43**), is a bisindole alkaloid derived from *Catharanthus roseus*. The apoptosis induced by cathachunine relied on the Bcl-2 protein family through an ROS- dependent mitochondria-mediated intrinsic pathway in HL60 cells. The ratio of Bcl-2/Bax was dysregulated, MMP was lost, cytochrome c was released, and production of ROS was increased [100].

A pyrrole based compound, Bis (2-ethyl hexyl) 1*H*-pyrrole-3, 4-dicarboxylate (**46**), from *Tinosporacordifolia* induced production of ROS, increased intracellular calcium levels, phosphorylated p53, downregulated Bcl-2/Bax ratio and led to cardiolipin peroxidation and mitochondrial membrane depolarization. Thereupon cytochrome c was released and caspases were activated, resulting in MDA-MB-231 cell apoptosis [101].

Unantimycin A (**47**), found in a fractionated chemical library of microbial metabolites, and NPL40330 (**48**), found in a chemical library, targeted and inhibited the activity of mitochondrial complexes I and III, respectively. Thus, they played a role in inhibiting mitochondrial respiration [102].

A 4-amido-2,4-pentadieneoate (APD)-class peptide named boholamide A (**49**) from a bacterial extract (*Nocardiopsis sp.*) from marine mollusks (*Truncatella sp.*), directly regulated intracellular Ca^2+^ in U87MG cells. Natural products of the APD-class have hypoxia-activated cytotoxins, targeting mitochondria [103]. Cernumidine (**50**) is a guanidinic alkaloid, which exerted antitumor effects through mitochondria by downregulating the Bcl-2/Bax ratio and causing MMP loss in the combination treatment with cisplatin in T24 cells [104]. Lycorine (**51**), extracted from plants of the *Amaryllidaceae* family, induced apoptosis in HepG2 cells through mPTP opening, ATP depletion, MMP loss, and mitochondrial Ca^2+^ and cytochrome c release [105]. Lagunamides A (**52**) from *Lyngbya majuscule* caused A549 cell death accompanied by MMP loss, ROS overproduction, mitochondrial dysfunction, and changes in the levels of Bcl-2 family proteins [106]. Cordycepin (**53**), isolated from Cordyceps, downregulated mitochondrial function and limited energy production, thus inhibiting metastasis and migration in OVCAR-3 cells [107,143].

### 5.5. Coumarins

**Figure 6:**
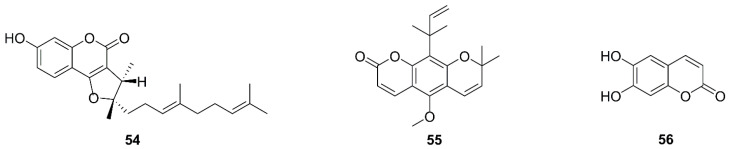
Chemical structures of coumarins (**54**–**56**).

Coumarin compounds are widespread in the plant kingdom, with a few coming from animals and microorganisms. Their basic backbone contains a fused benzene and α-pyrone ring [108]. They are widely present in Umbelliferae, Leguminosae, Rutaceae, Solanaceae, and Asteraceae [109] and are found in many traditional Chinese medicines. Coumarin has extensive pharmacological activities, such as anti-inflammatory, antihyperlipidemia, antihypertensive, and antitumor [110]. Chemical structures of coumarin in recent studies are displayed in Figure 6.

The 2,3-Dihydro-7-hydroxy-2R*,3R*-dimethyl-2-[4,8-dimethyl-3(*E*),7-nonadienyl]-furo[3,2-c]coumarin (**54**), named DAW22, is a sesquiterpene coumarin extracted from *Ferula ferulaeoides*. In C6 glioma cells, apoptosis induced by DAW22 is mediated by the death receptor pathway and mitochondrial pathway. It reduced MMP in a time-dependent manner. Results showed that the expression of Bax significantly increased, whereas that of Bcl-2 and Bcl-xL decreased, and the cleavage of Bid was stimulated. Moreover, the level of FAS (recombinant factor related apoptosis) and FADD (Fas-associated protein with death domain) were elevated markedly [111].

Dentatin (**55**), isolated from *Clausena excavate*, could increase the level of cytoplasmic cytochrome c and Bax, and down-regulate Bcl-2 and Bcl-xL in HepG2 cells [112,113]. Aesculetin (**56**), a natural coumarin derivative of intramolecular cyclization produced by a cinnamic acid exerted antitumor effects via mitochondrial mediated apoptosis in THP-1 macrophage cells with upregulating Bax and downregulating Bcl-2 [114].

### 5.6. Quinones

**Figure 7:**
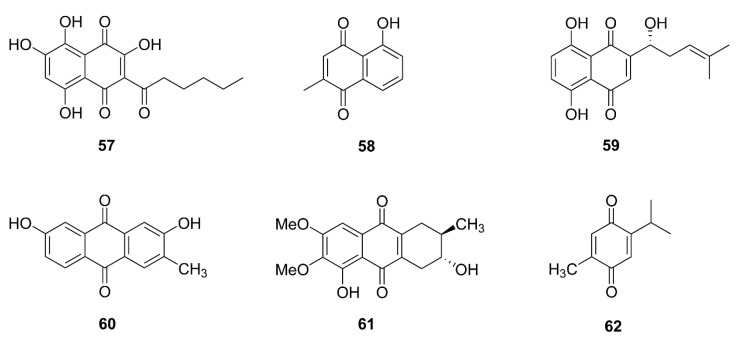
Chemical structures of quinines (**57**–**62**).

Natural quinones, the compounds containing a six-member cyclic conjugated unsaturated diketone structure, mainly include four types, benzoquinone, naphthoquinone, phenanthrenequinone, and anthraquinone. Anthraquinone and its derivatives are particularly important in traditional Chinese medicine. Chemical structures of quinines in recent studies are displayed in Figure 7.

Quambalarine B (**57**) is a natural naphthoquinonic compound from *Quambalariac yanescens* [115]. It inhibited the activity of mitochondrial complex I and II and reduced the metabolism of aspartic acid and folic acid as therapeutic targets in Jurkat cells. Inhibition of mitochondrial respiration by quambalarine B triggered a reprogramming of leukemic cell metabolism, including an imbalance of glycolysis, inhibition of protein o-glycosylation, increased activity of pyruvate kinase, and stimulation of glycine synthesis pathways, and inhibition of aspartate synthesis. This led to increased pyruvic acid and decreased lactic acid levels. To inhibit mitochondrial complex I activity, quambalarine B inhibits folic acid metabolism, reducing the production of formate. In addition, several amino acids were increased at the cellular level [116].

Plumbagin (**58**), a naphthoquinone, is derived from *Plumbago zeylanica*. After treatment with plumbagin in MG63 cells for 24 h, production of ROS was increased, the protein levels of Bcl-2, Bax, Bcl-xL, and Bak were altered [117]. The naphthoquinone pigments shikonin (**59**) of *Lithospermum erythrorhizon* had a similar mechanism in HGC-27 cells, like that of plumbagin in MG63 cells [118].

The 2,7-dihydroxy-3-methylanthraquinone (**60**), isolated from *Hedyotisdiffusa*, decreased the expression of Bcl-xL and Bcl-2, increased Bax and Bad, released cytochrome c, and activated caspase-3 and -9 in SGC-7901 cells [119]. Moreover, 3-hydroxy-1,5,6-trimethoxy-2-methyl-9,10-anthraquinone (**61**) derived from *Prismatomeris connate* reduced expression of Bcl-2 and Mcl-1, and increased Bax in A549 and H1299 cells [120]. They are similar to thymoquinone (**62**), a compound of the black seed oil from *Nigella sativa* [121].

### 5.7. Miscellanea

**Figure 8:**
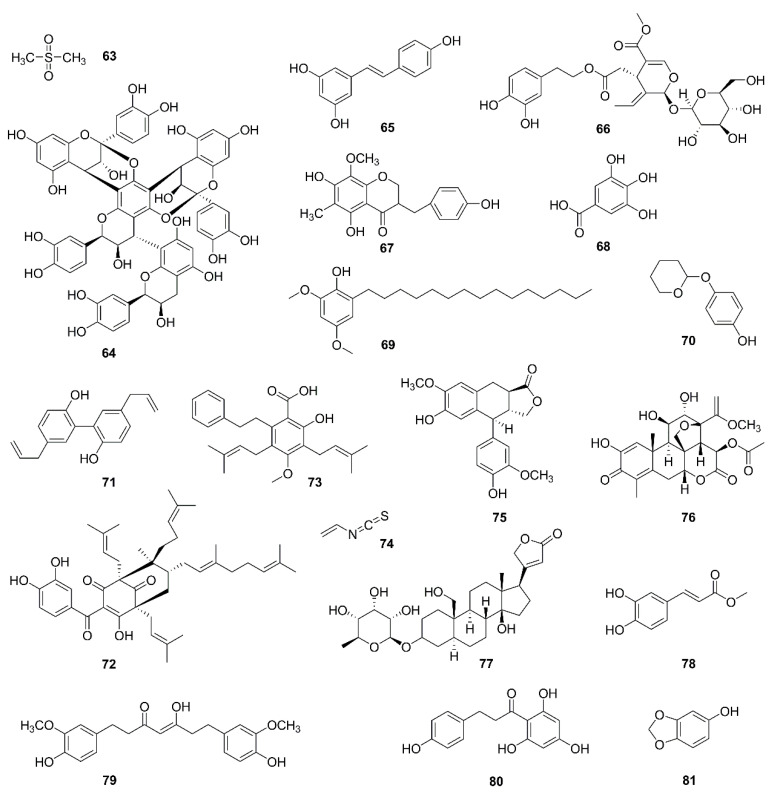
Chemical structures of other compounds isolated from natural products (**63**–**81**).

Other natural products, different from the above structures (Figure 8), had an antitumor effect by regulating mitochondria.

For example, interesting research found that macrocyclic lipodepsipeptides containing 4-amido-2,4-pentadienoate had cytotoxic selectivity for hypoxic cancer cells by inducing a rapid loss of mitochondrial ultrastructure and function [122].

Methylsulfonylmethane (**63**), a natural organic sulfur-containing compound found in fruits and vegetables, decreased Bcl-2 and Bcl-xL levels and MMP, increased Bax level, and released cytochrome c into the cytosol in YD-38 gingival cancer cells [123]. Recombinant buckwheat trypsin inhibitor, extracted from tartary buckwheat, induced mitophagy, depolarized mitochondria, and increased ROS in HepG2 cells [124].

Some phenols regulate mitochondria. Parameritannin A-2 (**64**), isolated from *Urceolahuaitingii,* enhances doxorubicin-induced mitochondria-dependent apoptosis in HGC27 gastric cancer cells, in part by inhibiting the PI3K/Akt, ERK1/2, and p38 pathways. After combination treatment with parameritannin A-2 and doxorubicin, protein levels of Bcl-2 and Bcl-xL were decreased, and Bax and Bid were increased more significantly than after the single treatments. Cytochrome c was released and caspases were activated [125]. Similarly, resveratrol (**65**) enhanced antitumor activities of cisplatin on H838 and H520 cancer cells [126]. Another study showed resveratrol induced apoptosis in K562 cells [127]. In addition, phenols like oleuropein (**66**) [128,129], homoisoflavanone-1 (**67**) [130], gallic acid (**68**) [131], hierridin b (**69**) [132,144], and deoxyarbutin (**70**) [145] could all induce mitochondrial dysfunction.

Magnolol (**71**), a phenylpropanoid, derived from *Magnolia officinalis* induced apoptosis in OS-RC-2 and 786-*O* cell lines by regulation of Bcl-2, Bax and p53. ROS generation, cytochrome c release and caspase activation were also observed [146].

Oblongifolin C (**72**), a polycyclic polyprenylated acylphloroglucinol (PPAP) compound, isolated from *Garcinia yunnanensis* induced mitochondrial dysfunction and apoptosis in QBC939 human cholangiocarcinoma cells [147].

Amorfrutin C (**73**), belongs to the amorfrutin benzoic acid class of compounds found in *Glycyrrhiza foetida*. Treatment with amorfrutin C disrupted the mitochondrial integrity and permanently opened mPTP, leading to increased mitochondrial oxygen consumption and extracellular acidification in HT-29 cells [148].

There are some other natural compounds exerting antitumor effect via mitochondria with ROS generation, cytochrome c release, MMP loss, altered expression of Bcl-2 family members, and caspase activation, such as allyl isothiocyanate (74) [149,150], α-conidendrin (**75**) [151], dehydrobruceine B (**76**) [152], frugoside (**77**) [153,154], methyl caffeate (**78**) [155], tetrahydrocurcumin (**79**) [156], phloretin (**80**) [157], and sesamol (**81**) [158].

## 6. Natural Products and Anticancer Agents in Combination

Natural products have attracted much attention because they are relatively easy to obtain and cause few side effects. Many scholars have studied their activities in combination with cancer drugs in vitro and vivo. Xia et al. showed that there was a protective effect of magnolol on oxaliplatin-induced intestinal injury in mice [159]. Magnolol significantly improved weight loss, diarrhea, and other adverse reactions after oxaliplatin administration.

In addition, some natural products can increase the activity of anticancer agents. They can be used—not only as a complementary and alternative therapy—but also to enhance the efficacy of resistant cell lines. Combination with cisplatin and chrysin promotes the apoptosis of HepG2 cells by upregulating p53 [160], polyphyllin I enhances apoptosis and suppresses the CIP2A/AKT/mTOR signaling pathway in A549/DDP cells [161]. Combining ginsenoside compound K with cisplatin produced a better effect on the apoptosis and epithelial mesenchymal transition through the PI3K/Akt pathway in MCF-7 cells [162]. The combined effect of α-Hederin and cisplatin was better than both compounds alone on apoptosis by increasing ROS and decreasing MMP in vitro and vivo [163]. Berberine [164], cernumidine [144], shikonin [165], gallic acid [131,166], and dehydrobruceine b [167] also had a chemosensitizing effect when added together with cisplatin.

Combination treatment of sorafenib and luteolin enhanced JNK activation and apoptosis in Hep3B and SMMC-7721 hepatocellular carcinoma cells [168]. Sorafenib and luteolin combination synergistically inhibited proliferation of AsPC-1, BxPC-3, and Capan-1 pancreatic ductal adenocarcinoma cells by targeting the P13K/Akt and MAPK signaling pathways [169]. Combination of α-mangostin and sorafenib enhanced apoptosis by inhibition of the activated Akt and Erk pathways in SK-MEL-2 cells and SK-MEL-30 cells [170].

Lupeol and 5-fluorouracil combination exerted a better effect on inhibition of tumor weight on BGC823 xenograft mouse [171]. Combination treatment with 5-fluorouracil and other natural compounds, such as fisetin [172], frondoside a [173], or esculetin [174] led to more significant effects on cancer cells. Similarly, there are highly efficacious co-treatments that use combinations of baicalein and taxol [81], thymoquinone and gemcitabine [175], parameritannin A-2 and doxorubicin [125], gallic acid and paclitaxel [176], and allyl isothiocyanate and celecoxib [177].

## 7. Conclusions

We focused on natural compounds that have been identified in the last five years with anticancer activity targeting mitochondria and their origin and structural classification. Phytochemistry accounts for most of the composition, with a small amount of marine and microbial metabolites. Most of these compounds are terpenoids, phenols, and flavonoids. Some of these compounds regulate the expression level of Bcl-2 family proteins, some induce ROS production, some alter metabolism, and some target mitochondrial complexes. This review shows that large and varied classes of plant-derived and other natural products can exert anticancer activity by regulating mitochondria. However, some natural products are non-toxic, show poor efficacy, or have special mitochondrial targeting, so it is of great significance to develop further structural modifications and derivatives based on the natural structures.

## Figures and Tables

**Table 1:** Natural products (**1–81**) regulated mitochondria by different mechanisms in cancer cells.

No.	Isolated Compound	Origin	Cell Line	Mechanism	Reference
*Terpenoids*					
**1**	Ganoleuconin O	*Ganoderma leucocontextum*	Huh7.5	Fatty acid immobilization, loss of the mitochondrial lipid cardiolipin	[30]
**2**	Lupeol	*Bombax ceiba*	SK-RC-45	Mitochondrial hyper fission	[31]
**3**	Betulinic acid	*Betula alba*	HeLa	Cardiolipin modification, ROS generation, Bad, caspase 9	[32,33]
**4**	Alisol B-23-acetate	*Alisma orientale*	A549, NCI-H292	ROS generation, Bcl-2↓, Bax↑, activation of caspase-3, -9, release of cytochrome c/AIF	[34]
**5**	Genipin	*Gardenia jasminoides*	N18TG2	Activation of dicarboxylate carrier, decreased activity of UCP1, UCP3, and complex III of the respiratory chain, UCP2 inhibition	[35]
**6**	Alternol	Yew tree	PC-3	Decrease of mitochondrial respiration, isocitric acid, fumaric acid and malic acid, ATP production	[36,37]
**7**	Cyathin Q	*Cyathus africanus*	HCT116	Bcl-2↓, Bax↑, Bcl-xL↓, ROS generation, release of cytochrome c	[38]
**8**	3α-hydroxy-19α-hydrogen-29-aldehyde-27-lupanoic acid	*Potentilla discolor*	HepG2	Bcl-2↓, Bax↑, release of cytochrome c	[39]
**9**	Uvedafolin	*Smallanthus sonchifolius*	HeLa	MMP loss, release of cytochrome c	[40]
**10**	Heteronemin	*Hippospongia* sp.	Molt4	ROS generation	[41]
**11**	Jatrogossone A	*Jatropha gossypiifolia*	KOPN-8	MMP loss, ROS generation	[42]
**12**	Walsuronoid B	*Walsura robusta*	Bel-7402, HepG2	ROS generation, mitochondrial and lysosomal dysfunction	[43]
**13**	Ferruginol	*Podocarpus ferruginea*	MDA-T32	ROS generation, MMP loss, Bcl-2↓	[44,45]
**14**	Lobocrassin B	*Lobophytum crassum*	CL1-5, H520, BEAS-2B	Bcl-2↓, Bax↑, ROS generation, MMP loss, release of cytochrome c, activation of caspase-3	[46]
**15**	Aellinane	*Euphorbia aellenii*	Caov-4	Bcl-2↓, Bax↑, ROS generation, MMP loss	[47]
**16**	Tingenin B	*Maytenus* sp.	MCF-7s	Bcl-2↓, Bax↑, MMP loss	[48]
**17**	3-*O*-*trans*-p-coumaroyl alphitolic acid	*Ziziphus jujuba*	PC-3	ROS generation	[49]
**18**	Zerumbone	*Zingiber zerumbet*	PC-3, DU-145	Tubulin binding and crosstalk between endoplasmic reticulum stress and mitochondrial insult	[50,51]
*Flavonoids*					
**19**	Isoquercitrin	*Hibiscus cannabinus*	MDA-MB-231	LSD1-induced mitochondrial-mediated apoptosis pathway	[52,53]
**20**	Luteolin	Cauliflower, peanut, and carrot	SW1990	Inhibitor of Bcl-2, mitochondrial permeabilization	[54]
**21**	Dihydromyricetin	*Ampelopsis grossedentata*	HepG2	Akt/Bad signal pathway, mitochondrial apoptotic pathway, Bax↑, Bad↑, inhibition of the phosphorylation of Bad at Ser136 and Ser112	[55,56]
**22**	Artonin E	*Artocarpus elasticus*	SKOV-3	Release of cytochrome c, Activation of caspases-3, -8, and -9, Bax↑, Bcl-2↓, HSP70↓, survivin↓	[57]
**23**	Myricetin	Fruits and vegetables	SNU-80	Bax/Bcl-2↑, release of AIF	[58]
**24**	Xanthones	*Garcinia xanthochymus*	HepG2	Bax↑, Bcl-2↓, Bcl-xL↓, Mcl-1↓, and survivin↓	[59]
**25**	Cycloartobiloxanthone	*Artocarpus gomezianus*	H460	Bax↑, Bcl-2↓, Mcl-1↓	[60]
**26**	Paratocarpin E	*Euphorbia humifusa*	MCF-7	Bax↑, Bcl-2↓, release of cytochrome c	[61]
**27**	Puerarin 6′’-*O*-xyloside	*Pueraria lobata*	SW480	Bax↑, Bad↑, Bcl-2↓, caspase-3 and -9 activation	[62]
**28**	α-mangostin	*Cratoxylum arborescens*	HeLa	ROS generation, MMP loss, release of cytochrome c	[63]
**29**	Chrysin	Honey and propolis	Mitochondria isolated from hepatocytes of HCC rats	ROS generation, MMP loss, release of cytochrome c, swelling in mitochondria	[64,65]
**30**	Fisetin	Strawberries, apples, grapes, onions, and cucumbers	SCC-4	ROS generation, Ca^2+^ production, MMP loss, Bcl-2↓, Bax↑, Bid↑, release of cytochrome c, AIF, and Endo G	[66,67]
**31**	Baicalein	*Scutellaria baicalensis*, *Scutellaria radix*	A2780	Combination therapy with baicalein and taxol had much higher antitumor effects compared with the monotherapy. Release of cytochrome c, and caspase-3 and -9 activation	[68,69]
**32**	Alpinetin	Zingiberaceous plants	A549	Bcl-2↓, Bax↑, Bcl-xL↓, XIAP↓, PI3K/Akt signaling pathway, sensitized drug-resistant lung cancer cells	[70,71]
**33**	Chamaejasmin B	*Stellerachamaejasme*	KB, KBV200	Bcl-2↓, Bax↑, MMP loss, release of cytochrome c and AIF	[72]
**34**	Mensacarcin	*Streptomyces* bacteria	SK-Mel-28, SK-Mel-5, HCT-116	Release of cytochrome c, energy production and mitochondrial function rapidly disturbed	[73]
*Saponins*					
**35**	Gracillin	*Dioscorea gracillima*	H226B, H460	Targeting mitochondrial complex II, suppressing ATP synthesis, ROS generation	[74]
**36**	Polyphyllin I	*Paris polyphylla*	MDA-MB-231	Mitochondrial translocation of DRP1, mitochondrial fission, release of cytochrome c, mitochondrial PTEN-induced kinase 1↑	[75,76]
**37**	Frondoside A	*Cucumaria frondosa*	CA46	Bcl-2↓, survivin↓, release of HtrA2/Omi and cytochrome c, ROS generation	[77]
**38**	3β-*O*-α-l- arabinopyranoside	*Clematis ganpiniana*	MCF-7, MDA-MB-231	Release of cytochrome c and Apaf-1, upregulation of caspase-9 and caspase-3	[78]
**39**	Sakuraso-saponin	*Aegiceras corniculatum*	LNcaP, 22RV-1, C4-2	Bcl-xL↓	[79,80]
**40**	Ginsenoside compound K	*Panax ginseng*	SK-N-BE(2), SH-SY5Y	Bcl-2↓, Bcl-xL↓	[81]
**41**	Escin	*Aesculus hippocastanum*	786-O, Caki-1	G2/M arrest and ROS-modulated mitochondrial pathways	[82]
**42**	α-Hederin	*Hedera helix*	SW620	NF-κB signaling pathway, Bcl-2↓, Bax↑, release of cytochrome c	[83,84]
*Alkaloids*					
**43**	Cathachunine	*Catharanthus roseus*	HL60	ROS-dependent mitochondria-mediated intrinsic pathway, Bcl-2/Bax↓, ROS generation, MMP loss, release of cytochrome c	[85]
**44**	Berberine	*Rhizoma coptidis*	T98G, LN18	ERK1/2-mediated impairment of mitochondrial aerobic respiration	[86,87]
**45**	Papuamine	*Haliclona* sp.	H1299	Intracellular ATP depleted by causing mitochondrial dysfunction, mitochondrial superoxide production	[88]
**46**	Bis (2-ethyl hexyl) 1*H*-pyrrole-3, 4-dicarboxylate	*Tinospora cordifolia*	MDA-MB-231	ROS generation, increase in intracellular calcium, phosphorylation of p53, mitochondrial membrane depolarization, MPTP, and cardiolipin peroxidation, Bcl-2↓, Bax↑, release of cytochrome c, caspase activation, DNA fragmentation	[89]
**47**	Unantimycin A	Found in the fraction library of microbial metabolites	Semi-intact cells with specific substrates for each complex of the mitochondrial electron transport chain	Targeted inhibition of mitochondrial complex I	[90]
**48**	NPL40330	Found in chemical library	Semi-intact cells with specific substrates for each complex of the mitochondrial electron transport chain	Targeted inhibition of mitochondrial complex III	[90]
**49**	Boholamide A	Marine mollusks	U87MG	Influx of Ca^2+^	[91]
**50**	Cernumidine	*Solanum cernuum*	T24	Cytotoxicity and chemosensitizing effect of cernumidine to cisplatin. Bcl-2↓, Bax↑, MMP loss	[92]
**51**	Lycorine	*Amaryllidaceae* plant family	HepG2	mPTP opening, MMP loss, ATP depletion, release of Ca^2+^ and cytochrome c, caspase activation	[93]
**52**	Lagunamides A	*Lyngbya majuscule*	A549	MMP loss, ROS generation	[94]
**53**	Cordycepin	Cordyceps	OVCAR-3	Downregulation of mitochondrial function and limitation of energy production; metastasis and migration suppressed	[95,96]
*Coumarins*					
**54**	2,3-Dihydro-7- hydroxy-2R*,3R*- dimethyl-2-[4,8-dimethyl-3(*E*),7- nonadienyl]-furo[3,2-c]coumarin	*Ferula ferulaeoides*	C6	MMP loss, Bcl-xL↓, Bcl-2↓, Bax↑, cleavage of Bid, FAS↑, FADD↑	[97]
**55**	Dentatin	*Clausena excavate*	HepG2	Bcl-xL↓, Bcl-2↓, Bax↑, release of cytochrome c	[98,99]
**56**	Aesculetin	*Cortex Fraxini*	THP-1	Bcl-2↓, Bax↑	[100]
*Quinones*					
**57**	Quambalarine B	*Quambalaria cyanescens*	Jurkat E6.1	Inhibition of mitochondrial complex I and II, inhibition of mitochondrial respiration, metabolism reprogramming	[101,102]
**58**	Plumbagin	*Plumbago zeylanica*	MG63	ROS generation, Bcl-2↓, Bax↑, Bcl-xL↓, and Bak↓, endoplasmic reticulum stress	[103]
**59**	Shikonin	*Lithospermum erythrorhizon*	HGC-27	Bcl-2↓, Bax↑, survivin↓	[104]
**60**	2,7-dihydroxy-3-methylanthraquinone	*Hedyotis diffusa*	SGC-7901	Bcl-xl↓, Bcl-2↓, Bax↑, Bad↑, release of cytochrome c	[105]
**61**	3-hydroxy-1,5,6-trimethoxy-2-methyl-9,10-anthraquinone	*Prismatomeris connate*	A549, H1299	Bcl-2↓, Mcl-1↓, Bax↑	[106]
**62**	Thymoquinone	*Nigella sativa*	T24, 253J	Bcl-2↓, Bax↑, release of cytochrome c and AIF	[107]
*Miscellanea*					
**63**	Methylsulfonylmethane	Fruits and vegetables	YD-38	Bcl-xL↓, Bcl-2↓, Bax↑, release of cytochrome c, MMP loss	[108,109]
**64**	Parameritannin A-2	*Urceola huaitingii*	HGC27	Enhanced doxorubicin-induced mitochondria-dependent apoptosis, inhibition of the PI3K/Akt, ERK1/2 and p38 pathways, Bcl-2↓, Bcl-xl↓, Bax↑, Bid↑, release of cytochrome c, caspase activation	[110]
**65**	Resveratrol	*Polygonum cuspidatum,* *Veratrum nigrum,* *Cassia obtusifolia*	H838, H520; K562	Enhanced antitumor activities of cisplatin; Induced apoptosis	[111,112]
**66**	Oleuropein	*Olea europaea*	H1299	Bcl-2/Bax↓, release of cytochrome c, activation of caspase-3	[113,114]
**67**	Homoisoflavanone-1	*Polygonatum odoratum*	A549	Mitochondria-caspase-dependent and ER stress pathways, Bcl-2/ Bak↓	[115]
**68**	Gallic acid	Green tea, grapes, red wine	H446	ROS-dependent mitochondrial apoptotic pathway	[116]
**69**	Hierridin b	*Cyanobium* sp.	HT-29	Proteomics identified 21 differentially expressed proteins belonging to the categories protein folding/synthesis and cell structure and reduced mitochondrial activity and as confirmed by morphological analysis of mitochondrial parameters	[117,118]
**70**	Deoxyarbutin	*Ecklonia cava*	B16F10	MMP loss, ATP depletion and ROS overload generation	[119]
**71**	Magnolol	*Magnolia officinalis*	OS-RC-2, 786-O	P53, Bcl-2/Bax↓, release of cytochrome c, caspase activation, ROS generation	[120]
**72**	Oblongifolin C	*Garcinia yunnanensis*	QBC939	Mitochondrial dysfunction	[121]
**73**	Amorfrutin C	*Glycyrrhiza foetida*	HT-29	mPTP opening, mitochondrial oxygen consumption and extracellular acidification increased	[122]
**74**	Allyl isothiocyanate	Cruciferous vegetables	MCF-7, MDA-MB-231	ROS and Ca^2+^ production, MMP loss, release of cytochrome c, AIF, and Endo G, Bcl-2↓, Bax↑	[123,124]
**75**	α-conidendrin	*Taxus yunnanensis*	MCF-7 and MDA-MB-231	ROS generation, p53↑, Bax↑, Bcl-2↓, MMP loss, release of cytochrome c, activation of caspases-3 and -9	[125]
**76**	Dehydrobruceine B	*Brucea javanica*	A549, NCI-H292	MMP loss, release of cytochrome c, cleavage of caspase-9, caspase-3, and poly (ADP-ribose) polymerase (PARP)	[126]
**77**	Frugoside	*Calotropis procera*	M14, A375	ROS generation	[127,128]
**78**	Methyl caffeate	*Solanum torvum*	MCF-7	Bcl-2↓, Bax↑, Bid↑, p53↑, cleavage of caspase-3 and PARP, release of cytochrome c	[129]
**79**	Tetrahydrocurcumin	*Curcuma longa*	MCF-7	ROS generation, Bcl-2↓, PARP↓, Bax↑, release of cytochrome c, MMP loss	[130]
**80**	Phloretin	Apple tree leaves and Manchurian apricot	EC-109	Bcl-2↓, Bax↑	[131]
**81**	Sesamol	Sesame seeds	HepG2	Bcl-2↓, Bax↑, MMP loss, H_2_O_2_ production, PI3K Class III/Belin-1 pathway	[132]
